# Prediction equation for estimating cognitive function using physical fitness parameters in older adults

**DOI:** 10.1371/journal.pone.0232894

**Published:** 2020-05-07

**Authors:** Gian Pietro Emerenziani, Maria Grazia Vaccaro, Giulia Izzo, Francesca Greco, Luca Rotundo, Roberto Lacava, Sandro La Vignera, Aldo Eugenio Calogero, Andrea Lenzi, Antonio Aversa

**Affiliations:** 1 Department of Experimental and Clinical Medicine, University of Catanzaro "Magna Græcia", Catanzaro, Italy; 2 Neuroscience Centre, Magna Graecia University, Catanzaro, Italy; 3 PST Umberto Primo, DSS CZ Ambulatorio Geriatria Disturbi Cognitive e Demenze, Catanzaro, Italy; 4 Department of Clinical and Experimental Medicine, University of Catania, Catania, Italy; 5 Department of Experimental Medicine, Policlinico Umberto I, Sapienza University of Rome, Rome, Italy; University of L’Aquila, ITALY

## Abstract

Ageing is associated with declines in cognitive functions and physical fitness (PF). Physical exercise training and physical activity (PA) have been shown to have positive effects on cognitive functions and brain plasticity. This study aims to establish a practical equation for evaluating cognitive functions using PF parameters in healthy older adults. One-hundred and two older subjects were physically and clinically evaluated. Participants performed the Short Physical Performance Battery (SPPB) and handgrip test (HG); general cognitive functions were examined using the Mini Mental State Examination (MMSE). For all of them, a multiple regression analysis was used to predict MMSE from age, SPPB and HG variables. The new equation was cross validated to determine its prediction accuracy. Considering that SPPB and MMSE reference score are not different between genders, only one equation was developed for females and males. Age, SPPB and HG correlated significantly (p<0.01) with the MMSE score. The developed equation was MMSE = 19.479 + (1.548 x SPPB)–(0.130 x age) (R^2^ = 0.72 and root mean square errors of 3.6). The results of PF are useful for exercise specialists to achieve the best physical exercise training and PA in older adults. In conclusion, this study showed for the first time that our new equation can be used to predict subjects’ cognitive functions based on SPPB results and subject age. We suggest its use when patients’ cognitive functions or more appropriate clinical tests cannot be pursued.

## Introduction

Between 2015 and 2050, the number of people over 60 will almost double from 12% to 22% [1]. As a result, the population structure will be changing in developed countries, with fewer children and more elderly people. Because of this change, the pace of population aging around the world is also increasing [1]. A longer life might bring new opportunities or disabilities depending on whether people can experience these additional years of life in a healthy or non-healthy condition. Indeed, if these added years are dominated by declines in physical and mental capacity the prevalence of many chronic conditions is expected to increase. Healthy ageing is defined as the process of developing and maintaining the functional ability that enables well-being in old age [1]. With increased longevity it is very important to prevent the age-related impairment of cognitive functions such as mild cognitive impairment (MCI) and dementia. Dementia, the end stage of brain diseases and Alzheimer disease (AD), is the most common one, while MCI is a heterogeneous state between normal ageing and early dementia. There are different methods to assess subjects’ cognition state, however, the most common one, employed in 80% of studies, is the Mini-Mental State Examination (MMSE) or its modified version [2]. A healthy lifestyle, including correct diet, abstinence from cigarette smoking and regular physical activity (PA) has a pivot role for healthy aging. Through the manuscript we use the term “physical activity” to indicate any bodily movements, “exercise” to indicate a subset of physical activity characterized by a planned and purposeful training, and “physical fitness” (PF) to indicate a set of attributes that are health related [3]. Many studies support the idea that PA might be considered as non-invasive therapy for physical and mental health improvements [4–6]. For instance, Blair et al. (1989) showed that high level of PF appears to delay all-cause mortality decreasing the rates of cardiovascular diseases and cancer [7]. It has been recognized that healthy lifestyle might counteract physical and cognitive decline in subjects affected by illness or impairment. PA has been recognized as the stronger factor to counteract the development of AD [8]. PA might maintain or improve cognitive functions in ageing and reduce the risk of AD in subjects older than 35 years old [9]. Moreover, physical active behaviors and PE might produce benefit in executive functions, and memory counteracting cognitive aging [10,11]. However, to reach these positive health effects, volume, intensity, frequency, and the type of exercise should be planned to achieve the best clinical results. When, these exercise parameters are not planned correctly, it could be possible that exercise might induce health complications or the training goals will not be achieved. Recent data suggest that functional mobility impairment in cognitive dual task is correlated to cognitive decline in patients with AD [12]. Dual-task actions require simultaneous motor and cognitive tasks and they are frequently used during daily living activity. Scientific research has been focused on the effects of dual-task training in older adults with [13] and without [14,15] mild-to moderate dementia and with Parkinson’s disease [16] showing an enhanced cognitive and physical functions after training. Moreover, Vaccaro et al. (2019) showed that dancing practice might increase fitness performance, memory functions and anxiety in older adults [17]. As previously reported, a decline in physical functions has been associated with cognitive decline [18]. Indeed, slow gait speed and weaker grip strength are strongly associated with worse cognitive performance [18]. Given that the evaluation of subjects’ physical functions is usually a non-invasive and well tolerated procedure, it could be useful to consider it as an additional marker for the assessment of MCI to validated expensive instrumental tests, i.e. positron emission tomography and functional magnetic resonance imaging. Moreover, whether it could be possible to estimate the global cognitive functions from physical fitness tests, sport science specialist could optimize the training program (e.g. choosing the most appropriate type of exercise) in order to counteract the subjects’ cognitive decline, using as example a dual-task training program. Therefore, the aim of this study was to establish a practical equation for evaluating cognitive functions using PF parameters in healthy older adults.

## Materials and methods

### Participants

One hundred and two older adults (65 females; 37 males) (age = 74.3±6.7 years, BMI = 28.3±4.0 kg/m^2^) were recruited in this study from patients admitted to Geriatric Evaluation Unit for Cognitive Disorders—Azienda Sanitaria Provinciale Catanzaro and Endocrinology Unit—Department of Experimental and Clinical Medicine, University Magna Graecia, Catanzaro. Inclusion criteria consisted of older age (> 65 years). The exclusion criteria were: physical impairment, severe psycho-cognitive diseases (major depressive disorder or psychosis), any neuropathy or autonomic dysfunction, significant renal or liver disease, uncontrolled cardiovascular disease, i.e., myocardial infarction/myocardial ischemia or ventricular tachycardia/obstructive valvular heart disease during the previous 6 months, uncontrolled hypertension (blood pressure values exceeding 140 mm Hg systolic or 90 mm Hg diastolic), uncontrolled hyperglycaemia, thyroid disease including autoimmunity, or any treatment with thyroid hormone preparations or amiodarone, methimazole or propylthiouracil in the prior 3 months. All participants underwent clinical examination to exclude any contraindications to PA and were recruited according to their willingness to participate to the study protocol and signature of informed consent. Moreover, independent samples of forty-five subjects (26 females; 19 males) (age = 78.4±6.4 years, BMI = 28.1±4.7 kg/m^2^) were selected for cross-validation analysis. These subjects were recruited using the same inclusion/exclusion criteria and from the same Centers. All tests were performed in the morning from 9:00 AM to 2:00 PM and MMSE was assessed with face-to-face interview by a trained physician. After that, subjects performed the Short Physical Performance Battery and Handgrip test in order to evaluate subjects’ physical fitness. Each participant provided a written informed consent before entering the study. This study was conducted according to the Declaration of Helsinki and was approved by the Ethical Committee of the Magna Graecia University, (approval number 149, 2017) as an amendment to baseline screening evaluation included in Eudract protocol n. 2016-005198-11.

### General cognitive functions, anthropometric and physical fitness assessment

Subjects’ general cognitive impairment were assessed by using the standardized neuropsychological Mini Mental State Examination (MMSE) test [19]. Height (to nearest 0.01 cm) and weight (to nearest 0.1 kg) were measured using a stadiometer with weighting station. Body mass index (BMI) was calculated dividing body weight in kilograms by height in meters squared (kg/m^2^). After a familiarization session, subjects performed the Short Physical Performance Battery (SPPB) [20] and Hangrip test (HG) [21]. The individual score of SPPB was derived from three functional tests that evaluate balance (Bal), lower body strength (CST) and gait speed (GS). The procedure is described in detail elsewhere [20]. Grip strength was measured using a JAMAR handheld dynamometer (BK-7498, Fred Sammons, Inc.) with participants seated, with their elbow by their side and flexed to right angles. The participants’ hand grip strength data were evaluated as left or right according to the dominant hand (the hand used in performing heavy tasks or using heavy tools). Subjects performed three trials and the average of the three attempts was used for data analysis. To minimize the effects of fatigue 45 seconds of recovery time was allowed between each trial.

### Statistical analysis

The Kolmogorov-Smirnov test was used to ensure normally distributed data. All data are presented as mean values ± standard deviation (SD). Differences between males and females were evaluated with an unpaired *t*-test. Correlation analysis was used to explore the relationships between MMSE and the physical fitness variables. Stepwise regression analysis was performed to identify which combination of significantly related variables would best predict MMSE measured by the interview. The coefficient of determination (R^2^) and the SEE were estimated. The criterion for inclusion (addition and retention) of predictors was the highest R^2^ model and the lowest SEE. Statistical significance was assumed at the conventional level of p ≤ 0.05. In the current study, cross-validation of predicted equations was performed by using the root mean squared error (RMSE) methods [22] to an independent sample. RMSE is a measure of the performance of prediction equation when applied to an independent sample. It is calculated as the square root of the sum of squared differences between the observed and the predicted values divided by the number of subjects in the cross-validation sample. All statistical analyses were performed with the SPSS statistical package (Version 24.0 for Windows; SPSS Inc., Chicago, IL, USA).

## Results

This section was divided by subheadings to provide a concise and precise description of the experimental results, their interpretation as well as the experimental conclusions that can be drawn.

### Cognitive functions, anthropometric and physical fitness results

Subjects’ cognitive functions, anthropometric characteristics and physical fitness results are shown in Table 1. As expected, height, weight, and HG were significantly higher (P < 0.01) in male than in female subjects. No differences were observed for age, body mass index (BMI), SPPB and MMSE variables between males and females (Table 1).

**Table 1:** Subjects’ anthropometrics characteristics, physical fitness and cognitive functions results. Data are presented as mean ± SD.

Parameters	Female (N = 65)	Male (N = 37)	Pooled (N = 102)	P value
Age (years)	73.4 ± 6.9	75.7 ± 6.1	74.3 ± 6.7	P = 0.10
Height (m)	1.52 ± 0.08	1.66 ± 0.07**	1.57 ± 0.09	P<0.01
Weight (kg)	66.5 ± 11.5	76.5 ± 11.1**	68.9 ± 12.7	P<0.01
BMI (kg/m)	28.6 ± 4.4	27.7 ± 3.2	28.3 ± 4.0	P = 0.27
CST (score)	2.9 ± 1.5	3.2 ± 1.1	3.0 ± 1.4	P = 0.28
GS (score)	2.8 ± 1.1	3.2 ± 1.0	3.0 ± 1.1	P = 0.10
Balance (score)	3.5 ± 0.9	3.5 ± 0.8	3.5 ± 0.9	P = 0.86
SPPB (score)	9.2 ± 3.1	9.9 ± 2.5	9.4 ± 2.9	P = 0.24
HG (score)	19.5 ± 6.8	26.4 ± 9.3**	22.0 ± 8.4	P<0.01
MMSE (score)	24.1 ± 6.4	25.1 ± 4.6	24.5 ± 5.8	P = 0.44

BMI = body mass index; CST = lower body strength; GS = gait speed; SPPB = Short Physical Performance Battery; HG = handgrip test; MMSE = Mini Mental State Examination;

** Statistically significant vs female (P < 0.01)

### Stepwise and multiple regression analyses between MMSE and independent variables

**Fig 1:**
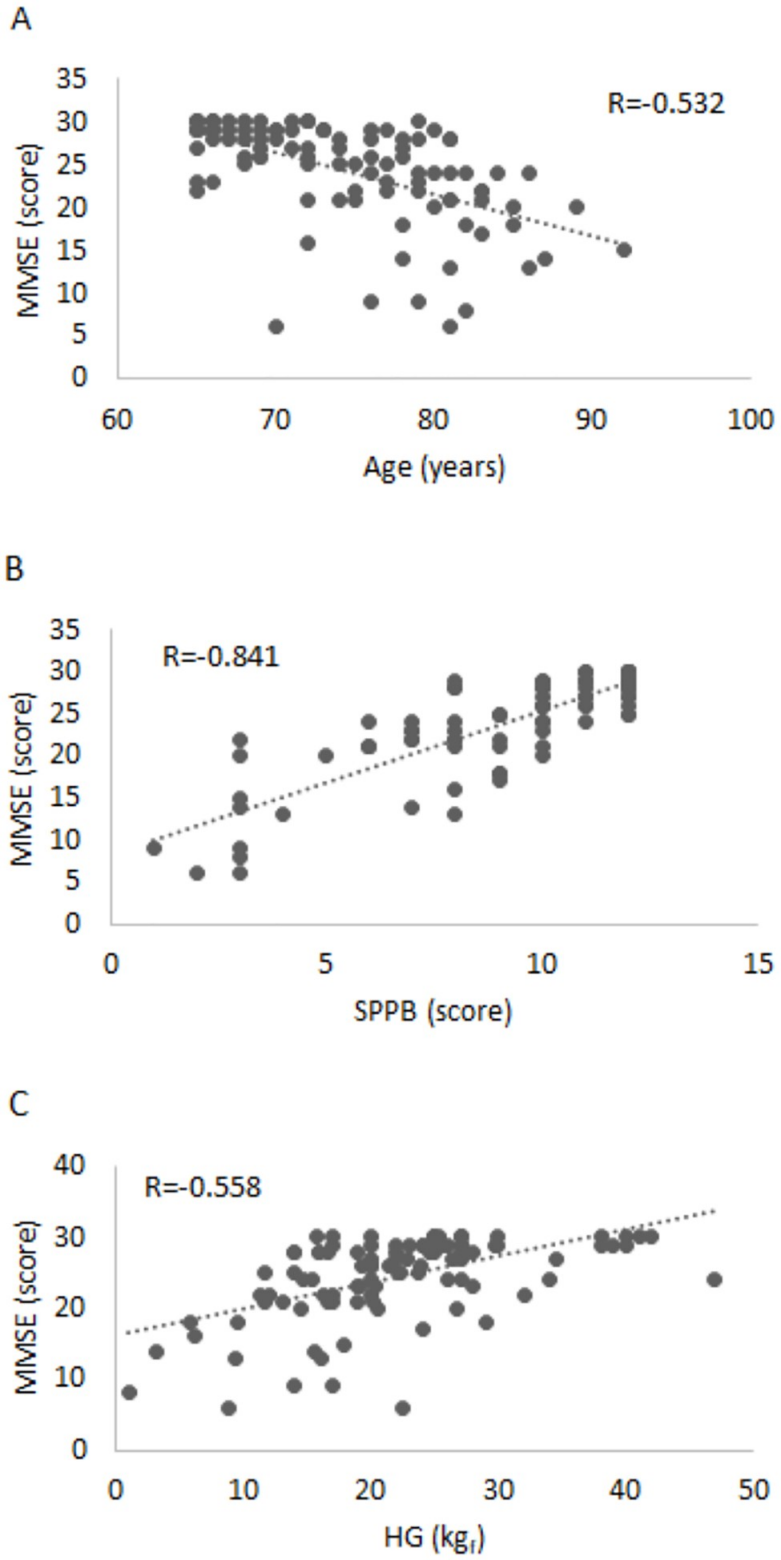
Correlation between Mini Mental State Examination (MMSE) score and age (a), Short Physical Performance Battery (SPPB) score (b) and Handgrip (HG) (c) in one hundred and two older adults (age range: 65 to 92 years).

Considering that SPPB and MMSE reference score are not different between genders, we decided to develop only one equation for both female and male subjects. MMSE showed significant (p<0.001) negative correlation with age (R = -0.532) (Fig 1a) and significant (p<0.001) positive correlation with SPPB (R = 0.841) (Fig 1b) and HG (R = 0.558) (Fig 1c).

The results of the stepwise multiple regressions showed that age, and SPPB data can give the best predictive model (R = 0.85, R^2^ = 0.72) as shown in Table 2.

**Table 2:** Stepwise regression analysis results.

Title 1	Coefficient	SE	R	SEE	P
Constant	19.479	4.812			
SPPB	1.548	0.127			
Age	-0.130	0.055			
Total model			0.850	3.1	<0.01

^1^ SPPB = Short Physical Performance Battery

From the result of multiple regression analysis, the prediction equations to estimate MMSE is:

MMSE=19.479+(1.548×SPPB)−(0.130×age)

For cross-validation analysis, prediction equations were used on forty-five subjects (age = 78.4 ± 6.4 years, SPPB = 9.0 ± 2.9 score. Subjects’ MMSE was 24.2 ± 5.6 score and predicted mean MMSE was 23.3 ± 4.9 score. The MMSE values for the RMSE were 3.0 score, therefore, RMSE was 13% of the range of target property value.

## Discussion

In this study we evaluated the correlation between PF parameters and MMSE score to establish a simple and practical equation that may help to predict cognitive functions in older adults. The results showed for the first time that, age and SPPB could be used as predictive variables of MMSE in older adults of both genders. It is worthy to mention that the subjects enrolled in our study were physically healthy, without any severe physical acute problem and severe psychological diseases that could negatively influence the results of PF tests. Moreover, we should point out the attention to the claim of our study that was not to establish a new method for MCI diagnosis; rather, we aimed to estimate subjects’ cognitive functions to prescribe the best PE protocols in older adults. In fact, more appropriate and validated methods are available for the evaluation of subjects’ cognitive functions, i.e. positron emission tomography and functional magnetic resonance imaging. With the increasing of population aging, it is important to apply any tool that could lead to a healthy aging such as be involved in PE practice. However, different PE parameters such as intensity, frequency and the type of exercise might influence the training effects. Therefore, the use of our practical equation might give to the sport science specialist more details in order to choose the most appropriate type of physical exercise (e.g. dual task exercise instead of strength exercise). Physical inactivity is associated with increased risk of cardiovascular and metabolic diseases that in turn are associated with increased risk of dementia [23]. Increased level of PA and PF result in a 20% lower mortality rate [24]. Moreover, Hu and colleagues (2004) showed that physically inactive middle-aged women have a 52% increase of all cause of mortality when compared with physically active subjects [25]. Korpelainen and co-workers (2016) reported that exercise capacity is the strongest predictor of cardiovascular diseases and all-causes of mortality in both genders especially for cardiovascular deaths in women [26]. Moreover, Myers et al. (2002) showed that each one metabolic equivalent (1 MET = 3.5 ml/kg/min) increase in exercise capacity conferred a 12% survival improvement in men [27]. Lower extremity muscle efficiency is also important in delaying the onset of disability since it correlates with gait and balance [28]. Falls are one of the causes of morbidity and mortality in older adults and gait and balance are also strongly associated with the risk of falls [28]. Once again, it has been shown that PE might prevent falls in community-dwelling older people [28]. However, PA and exercise do not have only positive effects on physical health but also on psychological well-being and cognitive functions, decreasing symptoms of anxiety and depression [5,29]. Indeed, it has well known that PA and PE may have a positive effect on cognition in multiple sclerosis [30], depression [31], stress disorders [32], and Parkinson’s disease [33]. The PE-related improvement in cognitive functions and psychological state seem to be associated with an increased expression of brain-derived neurotrophic factor (BDNF), glial cell-derived neurotrophic factor (GDNF) in some brain areas and insulin growth factor-1 (IGF-1) [34; 4]. BDNF is a growth factor expressed in the brain and throughout the rest of the central nervous system [35] and enhances the survival and differentiation of neurons, even dendritic arborization and synaptic plasticity [36]. Moreover, like BDNF, IGF-1 plays a fundamental role in many exercise-induced adaptations in the brain. The positive effects of PA on psychological state is also due to the increase of β-endorphin in peripheral blood resulted after exercise and it depends on the exercise intensity performed [37].

Low level of PF is linked to low cognitive performance and this relation could be explained by changes in the neurotrophic factors in the brain. Scientific literature showed that decreased physical performance is associated with poor cognitive functions [38]. Veronese and colleagues (2016) showed that slow walking speed precedes the onset of poor cognitive functions and that poor SPPB score is significantly associated with the onset of cognitive impairment in both genders. Moreover, chair standing time predicts the onset of cognitive impairment in females [38]. The authors [38], elucidated that one reason of the relation between gait speed and cognitive impairment should be that gait speed is closely associated with an impaired balance and fear of falling which has been associated with grey matter volume loss. Our results are in agreement with those reported by Veronese and co-workers; indeed, muscle strength and SPPB are positively correlated to MMSE showing that muscle strength and PF are correlated to subjects’ cognitive functions. However, to reach these positive health and physiological effects, volume, intensity, frequency and the type of exercise should be planned to achieve the best clinical results.

Scientific evidence showed that both endurance and resistance training may lead to positive results on subjects’ physical health by decreasing the risk of fall and by increasing the cardiovascular capacity and cognitive functions in older people [13,28,39]. Indeed, it is known that resistance-exercise training improves cognitive functions in healthy older adults [40] and that the types of PA might influence differently the structural and functional brain [41]. Recently, the number of studies on the effects of physical-cognitive dual task training on cognitive functions has increased [14,42]. Dual-task exercise requires a multitasking ability since subjects must simultaneously perform two tasks (physical and cognitive). For instance, subjects might walk while processing a cognitive task (e.g. counting backwards) simultaneously. As previously reported by Falbo and colleagues (2016) the addition of dual-task exercise to physical training enhance gait performance in general, suggesting to include dual-task exercise into physical training. To date, no equation allowing estimation of MMSE from SPPB and age in older adults has been published. The possibility to estimate the subjects’ cognitive level in older adults might lead the physical exerciser specialists to choose the best training protocol to reach the greatest clinical positive effects. Our regression model might be useful and suitable to all professionals that work in interdisciplinary teams to realize and optimize PE intervention. Our results have shown that SPPB was the highest predictor of MMSE whit a correlation coefficient equal to 0.841. The second predictor was people age with a correlation coefficient of 0.532. As expected, PF (SPPB) and age variables are strongly correlated with MMSE. In fact, a high level of PF resulted in a positive while age in a negative relationship with MMSE, respectively. A lower SPPB score and higher age will result in a low MMSE, on the contrary, higher SPPB score and younger age will result in a high MMSE score. The standard error of our predicted equation was 3.1. As described by Alexander and co-workers (2015) [22], the coefficient of determination (R^2^), the value of the root mean squared error (RMSE), and the use of an independent test set are recommended to characterize the external predictively of the model. In detail, values of R^2^ > 0,6 and MRSE < 10% are suggested [22]. In our equation, R^2^ value was 0.72 and RMSE value from the cross-validation results was 13% of the range of target property values. Although the well-known and more invasive (positron emission tomography) and expensive test (functional magnetic resonance imaging) remain the gold standard method for the assessment of cognitive impairments, this study suggests a valid alternative and an easier method to estimate MMSE when these methods are not available.

Nevertheless, when using this equation researchers and exercise professionals should be cautious to exclude older adult with physical and psychological diseases that might influence the SPPB results. We are aware of some important study limitations. For instance, different physical tests including other variables may be used for future studies to establish a new equation to estimate MMSE with RMSE lower than 10%. In addition, in our study the evaluation of subjects’ cognitive level was not supported with diagnostic imaging tests. Moreover, our cohort was made up of physical healthy subjects without severe psychological disease that could influence the PF tests and that could be able to attend an exercise training program. For all these reasons, future studies may implement the current equation with new parameters and different healthy subjects’ variables to achieve the highest correlation.

### Conclusions

This is the first study aimed to establish a practical reference equation to estimate the MMSE in healthy older adults. SPPB and age might be used to predict MMSE in both genders. This practical reference equation may a valid and alternative method to evaluate the cognitive functions in elderly when gold standard methods are not applicable or available in clinical practice and it could be useful to the sport science specialist in order to choose the most appropriate type of exercise training.

Finally, this design suggests several clinical research perspectives. In the next studies it will be interesting to evaluate if there are different levels of oxidative stress [43] capable of interfering on the validation of this equation and contextually evaluate the correlation with the quality of sexual activity [44], as well as compare aerobic exercise patterns vs other types of exercise.

## Supporting information

S1 File (PDF)

## References

[1] WHO 2- World Health Organization. 1st World Report on Ageing and Health, WHO. Geneva: WHO, 2015. https://www.who.int/ageing/events/world-report-2015-launch/en/

[2] Demnitz N, Esser P, Dawes H, Valkanova V, Johansen-Berg H, Ebmeier KP, et al A systematic review and meta-analysis of cross-sectional studies examining the relationship between mobility and cognition in healthy older adults. Gait Posture 2016, 50, 164–174, 10.1016/j.gaitpost.2016.08.028 27621086 PMC5081060

[3] Caspersen CJ, Powell KE, Christenson GM. Physical activity, exercise, and physical fitness: definitions and distinctions for health-related research. Public Health Rep 1985, 100(2), 126–131. 3920711 PMC1424733

[4] Vina J, Sanchis-Gomar F, Martinez-Bello V, Gomez-Cabrera MC. Exercise acts as a drug; the pharmacological benefits of exercise. Br J Pharmacol 2012, 167(1), 1–12, 10.1111/j.1476-5381.2012.01970.x 22486393 PMC3448908

[5] Ruegsegger GN, Booth FW. Health Benefits of Exercise. Cold Spring Harb Perspect Med 2018, 8(7), pii: a029694, 10.1101/cshperspect.a029694 28507196 PMC6027933

[6] Macera CA, Hootman JM, Sniezek JE. Major public health benefits of physical activity. Arthritis Rheum 2003, 49(1), 122–128, 10.1002/art.10907 12579603

[7] Blair SN, Kohl HW 3rd, Paffenbarger RS Jr, Clark DG, Cooper KH, Gibbons LW. Physical fitness and all-cause mortality. A prospective study of healthy men and women. JAMA 1989, 262(17), 2395–2401, 10.1001/jama.262.17.2395 2795824

[8] Barnes DE, Yaffe K. The projected effect of risk factor reduction on Alzheimer’s disease prevalence. Lancet Neurol 2011, 10(9), 819–828, 10.1016/S1474-4422(11)70072-2 21775213 PMC3647614

[9] Ngandu T, Lehtisalo J, Solomon A, Levälahti E, Ahtiluoto S, Antikainen R, et al A 2 year multidomain intervention of diet, exercise, cognitive training, and vascular risk monitoring versus control to prevent cognitive decline in at-risk elderly people (FINGER): a randomised controlled trial. Lancet 2015, 385(9984), 2255–2263, 10.1016/S0140-6736(15)60461-5 25771249

[10] Young J, Angevaren M, Rusted J, Tabet N. Aerobic exercise to improve cognitive functions in older people without known cognitive impairment. Cochrane Database Syst Rev 2015, (4):CD005381, 10.1002/14651858.CD005381.pub4 25900537 PMC10554155

[11] Hillman CH, Erickson KI, Kramer AF. Be smart, exercise your heart: exercise effects on brain and cognition. Nat Rev Neurosci 2008, 9(1), 58–65, 10.1038/nrn2298 18094706

[12] Borges SM, Radanovic M, Forlenza OV. Correlation between functional mobility and cognitive performance in older adults with cognitive impairment. Neuropsychol Dev Cogn B Aging Neuropsychol Cogn 2018, 25(1), 23–32, 10.1080/13825585.2016.1258035 27934540

[13] Chen YL, Pei YC. Musical dual-task training in patients with mild-to-moderate dementia: a randomized controlled trial. Neuropsychiatr Dis Treat 2018, 14, 1381–1393, 10.2147/NDT.S159174 29881275 PMC5985768

[14] Falbo S, Condello G, Capranica L, Forte R, Pesce C. Effects of Physical-Cognitive Dual Task Training on Executive Function and Gait Performance in Older Adults: A Randomized Controlled Trial. Biomed Res Int 2016, 2016:5812092, 10.1155/2016/5812092 28053985 PMC5178854

[15] Norouzi E, Vaezmosavi M, Gerber M, Pühse U, Brand S. Dual-task training on cognition and resistance training improved both balance and working memory in older people. Phys Sportsmed 2019, 47(4), 471–478, 10.1080/00913847.2019.1623996 31155997

[16] Yogev-Seligmann G, Giladi N, Brozgol M, Hausdorff JM. A training program to improve gait while dual tasking in patients with Parkinson’s disease: a pilot study. Arch Phys Med Rehabil 2012, 93(1), 176–181, 10.1016/j.apmr.2011.06.005 21849167

[17] Vaccaro MG, Izzo G, Ilacqua A, Migliaccio S, Baldari C, Guidetti L, et al Characterization of the Effects of a Six-Month Dancing as Approach for Successful Aging. Int J Endocrinol 2019, 2019:2048391, 10.1155/2019/2048391 31316562 PMC6601485

[18] Hooghiemstra AM, Ramakers IHGB, Sistermans N, Pijnenburg YAL, Aalten P, Hamel REG, et al Gait Speed and Grip Strength Reflect Cognitive Impairment and Are Modestly Related to Incident Cognitive Decline in Memory Clinic Patients With Subjective Cognitive Decline and Mild Cognitive Impairment: Findings From the 4C Study. J Gerontol A Biol Sci Med Sci 2017, 72(6), 846–854, 10.1093/gerona/glx003 28177065

[19] Measso G, Cavarzeran F, Zappalà G, Lebowitz BD, Crook TH, Pirozzolo FJ, et al The mini‐mental state examination: Normative study of an Italian random sample. Developmental Neuropsychology, 1993, 9:2, 77–85, 10.1080/87565649109540545

[20] Guralnik JM, Simonsick EM, Ferrucci L, Glynn RJ, Berkman LF, Blazer DG et al A short physical performance battery assessing lower extremity functions: association with self-reported disability and prediction of mortality and nursing home admission. J Gerontol 1994, 49, 85–94, 10.1093/geronj/49.2.m85 8126356

[21] Wang CY, Chen LY. Grip strength in older adults: test-retest reliability and cut off for subjective weakness of using the hands in heavy tasks. Arch Phys Med Rehabil 2010, 91(11), 1747–1751, 10.1016/j.apmr.2010.07.225 21044721

[22] Alexander DL, Tropsha A, Winkler DA. Beware of R(2): Simple, Unambiguous Assessment of the Prediction Accuracy of QSAR and QSPR Models. J J Chem Inf Model 2015, 55(7), 1316–1322, 10.1021/acs.jcim.5b00206 26099013 PMC4530125

[23] Profenno LA, Porsteinsson AP, Faraone SV. Meta-analysis of Alzheimer’s disease risk with obesity, diabetes, and related disorders. Biol Psychiatry 2010, 67(6), 505–512, 10.1016/j.biopsych.2009.02.013 19358976

[24] Myers J, Kaykha A, George S, Abella J, Zaheer N, Lear S, et al Fitness versus physical activity patterns in predicting mortality in men. Am J Med 2004, 117(12), 912–918, 10.1016/j.amjmed.2004.06.047 15629729

[25] Hu FB, Willett WC, Li T, Stampfer MJ, Colditz GA, Manson JE. Adiposity as compared with physical activity in predicting mortality among women. N Engl J Med 2004, 351(26), 2694–2703, 10.1056/NEJMoa042135 15616204

[26] Korpelainen R, Lämsä J, Kaikkonen KM, Korpelainen J, Laukkanen J, Palatsi I, et al Exercise capacity and mortality—a follow-up study of 3033 subjects referred to clinical exercise testing. Ann Med 2016, 48(5), 359–366, 10.1080/07853890.2016.1178856 27146022

[27] Myers J, Prakash M, Froelicher V, Do D, Partington S, Atwood JE. Exercise capacity and mortality among men referred for exercise testing. N Engl J Med 2002, 346(11), 793–801, 10.1056/NEJMoa011858 11893790

[28] Sherrington C, Michaleff ZA, Fairhall N, Paul SS, Tiedemann A, Whitney J, et al Exercise to prevent falls in older adults: an updated systematic review and meta-analysis. Br J Sports Med 2017, 51(24), 1750–1758, 10.1136/bjsports-2016-096547 27707740

[29] Kujala UM. Born to be rich, physically active, fit and healthy? Scand J Med Sci Sports 2010, 20(3):367, 10.1111/j.1600-0838.2010.01137.x 20598095

[30] Beier M, Bombardier CH, Hartoonian N, Motl RW, Kraft GH. Improved physical fitness correlates with improved cognition in multiple sclerosis. Arch Phys Med Rehabil 2014, 95(7), 1328–1334, 10.1016/j.apmr.2014.02.017 24607835

[31] Mura G, Moro MF, Patten SB, Carta MG. Exercise as an add-on strategy for the treatment of major depressive disorder: A systematic review. CNS Spectr 2014, 19(6), 496–508, 10.1017/S1092852913000953 24589012

[32] Schoenfeld TJ, Rada P, Pieruzzini PR, Hsueh B, Gould E. Physical exercise prevents stress-induced activation of granule neurons and enhances local inhibitory mechanisms in the dentate gyrus. J Neurosci 2013, 33(18), 7770–7777, 10.1523/JNEUROSCI.5352-12.2013 23637169 PMC3865561

[33] Mattson MP. Interventions that improve body and brain bioenergetics for Parkinson’s disease risk reduction and therapy. J Parkinsons Dis 2014, 4(1), 1–13, 10.3233/JPD-130335 24473219

[34] Liu PZ, Nusslock R. Exercise-Mediated Neurogenesis in the Hippocampus via BDNF. Front Neurosci 2018, 12:52, 10.3389/fnins.2018.00052 29467613 PMC5808288

[35] Salehi A, Delcroix JD, Mobley WC. Traffic at the intersection of neurotrophic factor signaling and neurodegeneration. Trends Neurosci 2003, 26(2), 73–80, 10.1016/S0166-2236(02)00038-3 12536130

[36] Park H, Poo MM. Neurotrophin regulation of neural circuit development and functions. Nat Rev Neurosci 2013, 14(1), 7–23, 10.1038/nrn3379 23254191

[37] Schwarz L, Kindermann W. Changes in beta-endorphin levels in response to aerobic and anaerobic exercise. Sports Med 1992, 13(1), 25–36, 10.2165/00007256-199213010-00003 1553453

[38] Veronese N, Stubbs B, Trevisan C, Bolzetta F, De Rui M, Solmi M, et al What physical performance measures predict incident cognitive decline among intact older adults? Exp Gerontol 2016, 81, 110–118, 10.1016/j.exger.2016.05.008 27235850

[39] Angevaren M, Aufdemkampe G, Verhaar HJ, Aleman A, Vanhees L. Physical activity and enhanced fitness to improve cognitive functions in older people without known cognitive impairment. Cochrane Database Syst Rev 2008, (3):CD005381, 10.1002/14651858.CD005381.pub3 18425918

[40] Chang YK, Pan CY, Chen FT, Tsai CL, Huang CC. Effect of resistance-exercise training on cognitive functions in healthy older adults: a review. J Aging Phys Act 2012, 20(4), 497–517, 10.1123/japa.20.4.497 22186664

[41] Voelcker-Rehage C, Niemann C. Structural and functionsal brain changes related to different types of physical activity across the life span. Neurosci Biobehav Rev 2013, 37(9 Pt B), 2268–2295, 10.1016/j.neubiorev.2013.01.028 23399048

[42] Wollesen B, Voelcker-Rehage C. Training effects on motor-cognitive dual-task performance in older adults: a systematic review. Eur Rev Aging Phys Act 2014, 11, 5–24.

[43] Sessa F, Messina G, Russo R, Salerno M, Castruccio Castracani C, Distefano A, et al Consequences on aging process and human wellness of generation of nitrogen and oxygen species during strenuous exercise [published online ahead of print, 2018 Jun 27]. Aging Male. 2018;1–9. 10.1080/13685538.2018.1482866 29950140

[44] Duca Y, Calogero AE, Cannarella R, Giacone F, Mongioi LM, Condorelli RA, et al Erectile dysfunction, physical activity and physical exercise: Recommendations for clinical practice. Andrologia. 2019;51(5):e13264 10.1111/and.13264 30873650

