# Supplementary material for: Prediction equation for estimating cognitive function using physical fitness parameters in older adults
Source: PLoS One. 2020 May 7;15(5):e0232894. doi: 10.1371/journal.pone.0232894 (PMC7205244; doi:10.1371/journal.pone.0232894)
Supplement: S1 File — (PDF) [file pone.0232894.s001.pdf]

Subject	Age	SPPB	MMSE	HG
1	70	2	6	22,4
2	81	3	6	8,9
3	82	3	8	1
4	76	1	9	17
5	79	3	9	14
6	86	4	13	9,3
7	81	8	13	16,1
8	78	3	14	3,2
9	87	7	14	15,5
10	83	9	17	24
11	92	3	15	17,9
12	72	8	16	6,2
13	78	9	18	5,8
14	85	9	18	29
15	82	9	18	9,6
16	74	8	21	17
17	80	10	20	26,8
18	77	7	22	32
19	66	8	23	19
20	83	3	22	11,3
21	89	3	20	14,5
22	72	10	21	20,2
23	79	9	22	20
24	75	6	21	16,6
25	79	10	23	20,4
26	85	5	20	20,6
27	65	8	22	17
28	81	9	21	19
29	81	6	21	11,7
30	83	6	21	13
31	77	7	23	28
32	75	7	22	12
33	65	7	23	19,1
34	76	10	24	34
35	80	6	24	26
36	82	10	24	20
37	68	10	26	21,4
38	83	8	22	16,3
39	79	10	24	27
40	68	9	25	14
41	86	7	24	14,7
42	72	12	25	22,3
43	74	9	25	11,7
44	78	11	26	19,2
45	69	10	26	20
46	81	11	24	47
47	84	8	24	15,4
48	75	12	25	22,2
49	65	12	27	22,8

50	69	12	28	19
51	72	12	26	20
52	78	12	27	34,6
53	71	11	27	27
54	76	10	26	20
55	74	11	28	16
56	69	10	27	22
57	77	9	25	23,8
58	67	12	28	22
59	66	8	28	14
60	67	11	29	20
61	65	11	29	27
62	66	12	29	25
63	72	11	27	20
64	74	11	27	26,4
65	79	10	28	14
66	69	11	26	24
67	70	12	28	28
68	70	12	29	24
69	73	12	29	30
70	68	10	28	16
71	78	12	28	27
72	68	10	29	40
73	66	12	29	38
74	65	12	29	22
75	81	8	28	16,7
76	81	12	28	24,5
77	69	12	30	42
78	77	12	29	39
79	76	12	29	24,7
80	73	11	29	24
81	76	12	28	25
82	71	11	30	16,9
83	72	11	30	20
84	72	12	30	25
85	72	12	30	25
86	69	12	29	17
87	65	12	29	26
88	66	12	30	41
89	65	12	30	27
90	68	11	30	25,3
91	80	12	29	27
92	66	10	29	23
93	70	12	29	25,5
94	73	12	29	27
95	71	8	29	29,8
96	79	12	30	15,8
97	65	12	30	25
98	65	12	30	27
99	66	12	30	27

100	67	12	30	30
101	66	12	30	40
102	65	12	30	38
103	69	12	28	25,6
104	72	12	30	30,0
105	71	11	30	25,6
106	71	12	30	30,0
107	76	12	29	32,0
108	75	8	28	38,0
109	85	12	29	26,0
110	74	11	30	24,6
111	81	10	29	13,7
112	76	12	30	36,2
113	78	12	30	14,0
114	89	12	30	28,4
115	84	7	18	28,4
116	73	11	26	34,0
117	81	10	28	28,7
118	84	5	16	18,9
119	73	12	27	38,2
120	76	2	12	16,7
121	83	9	20	20,2
122	85	9	27	19,1
123	84	8	16	23,7
124	68	12	28	17,9
125	79	5	14	17,7
126	78	10	25	32,4
127	83	4	19	15,1
128	89	3	10	7,3
129	82	5	20	16,8
130	88	12	27	16,6
131	71	9	25	20,0
132	74	9	28	27,0
133	80	8	28	16,8
134	83	5	20	14,9
135	87	11	29	31,1
136	77	9	20	19,6
137	65	11	28	52,1
138	82	9	23	40,2
139	84	11	23	24,8
140	67	12	29	39,9
141	76	10	26	16,7
142	88	6	19	19,0
143	78	8	19	19,9
144	76	3	20	15,0
145	77	9	17	23,1
146	68	11	29	30,6
147	86	6	18	11,7
